# Sewage and Sewer Gases

**Published:** 1858-10

**Authors:** H. Letheby


### SEWAGE AND SEWER GASES.

[From Dr. LETHEBY'S *Report to the Commissioners of Sewers of the City of London*, on Tuesday, September 14th, 1858.]

IN the present article, we shall give in his own words all the more important facts recorded by Dr. Letheby in his report. The subject discussed, as one of great interest at the present time, could not have been more happily expounded for the people than in the paper from which we copy.

"THE NATURE OF SEWAGE. The matters to be dealt with in the public sewers of every town and city are very complex, for they are composed not only of the solid and liquid ejecta of the population, but also of the fluid refuse of every branch of industry. They consist of the filth of kitchens, laundries, and dye-houses; the drainings from stables, slaughter-houses, and the public markets; the various liquid impurities of trade and manufactures; and the washings of the streets and alleys: all of which, with the ejecta of the inhabitants, and a large quantity of water, compose the sewage of towns. Each of these constituents, however, has its influence on the composition of the general mass, and on the putrefaction to which it is liable. Every part of a city, therefore, has its own peculiar quality of sewage—the quality being affected by the density of the population; the habits of the people, as to their diet, cleanliness, and trade pursuits; by the season of the year; the day of the week; the hour of the day; and even the state of the weather. This makes it difficult to obtain precise information of the nature and composition of sewage. Nevertheless, there are two ways by which a knowledge of the subject may be approached, viz., by the synthetical method of ascertaining the average amount of solid and liquid matter contributed by each of the inhabitants; and, secondly, by the analytical process of examining the sewage at different places, and at different times and seasons. Both of these plans of research are, no doubt, open to objection, but still they give results which are sufficiently accurate for our present purpose.

"The first method of inquiry has been pursued by Mr. Lawes, and by Professor Way; and the results of their investigations are that from 2 to $2\frac{1}{4}$ ounces of dry solid matter are contained in the excrements, *per diem*, of each member of the population. In the moist state as they are discharged from the body, they amount to about forty ounces *per diem*. These give a daily total of 152·6 tons of dry matter, or 2993·6 of moist, for the excrements of the whole population of London at the present time. But, while on the one hand, the ejecta are not entirely discharged into the sewers, so on the other, the numbers take no account of the contributions from trade, or of the washings from streets. I am not acquainted with any data for the estimation of the first of these, but the second may be ascertained approximatively from the analytical results of

Professor Way. He has found that the rain-water which runs from the streets into the gullies after a heavy shower of rain, contains about 262·6 grains of solid matter per gallon, of which 113·34 are in solution, and 149·26 in suspension. In the case of the granite roads, where the traffic is very large, the total amount of solid matter is 813·33 grains per gallon, of which 276·23 are dissolved, and 537·1 suspended; and in that of the wood pavements with the same kind of traffic, the amount is only 39 grains per gallon, of which 34 are dissolved, and 5 suspended. It will be manifest from this, that a large quantity of solid matter is carried from the streets into the public sewers during heavy rains. Altogether, it may be said, that the ejecta of the inhabitants of this metropolis, and the washings of the streets daily furnish about 233 tons of solid matter to the sewage; and these, with the trade refuse, are diluted with about 84¾ million gallons of water.

"The second mode of estimating the composition of sewage is founded on the analytical results of its examination at different times and places. These results show that the sewage which is discharged by day from the city sewers, contains about 94 grains of solid matter per gallon, of which 38 are suspended, and 56 dissolved: of the suspended, 17 are organic, and 21 mineral; and of the dissolved, 15 are organic and 41 mineral. The night sewage is not so rich in solid elements, for it contains only about 79 grains of solid matter per gallon; of which 14 are suspended, and 65 dissolved; and of these 15 are organic, and 64 mineral, the organic being distributed very evenly between the soluble and insoluble constituents.

"The sewage of the Tower Dock sewer contains by far the largest proportion of solid matter. A gallon of it furnishes about 921 grains of solid impurity; of which about 890 grains are in solution. The chief constituent is common salt,—a trade refuse of the neighbouring fishmongers. That which has the least impurity is the sewage which flows into the Whitefriars Dock, for it contains only about 41 grains of solid matter per gallon; of which 14·5 are suspended, and 26·5 dissolved.

"Branch sewers, and those which are nearly stagnant, are generally very foul; for the sewage of them contains from 150 to 500 grains of solid matter per gallon; of which from 90 to 250 are suspended. The organic matter ranges from 20 to 120 grains in the soluble part, and from 20 to 176 in the insoluble.

"Taking the average of all the results obtained in the examination of the metropolitan sewers, it may be concluded that the sewage which flows into the Thames contains about 90¼ grains of solid matter in the gallon; of which about 29¾ are suspended, and 60½ dissolved: there being about 15 grains of organic matter in each of these constituents.

"As I have already said, a storm of rain does not diminish the proportion of solid matter; for although it has a tendency to dilute the sewage, yet it washes away so large a quantity of filth from the streets, and disturbs so much of the sediment in the stagnant sewers, that sewage after a storm generally contains more than the average proportion of solid matter. Taking 88 grains per gallon as the usual amount in some of the sewers, it will, after a storm, be increased to 125 grains per gallon; of which 64 will be suspended, and 61 dissolved. So that the quantity of filth removed by a heavy fall of rain is enormous.

"The mineral constituents of sewer water are chiefly carbonate of lime and common salt, with small proportions of the alkaline sulphates and phosphates. They are derived from urine and from the water supply. The mineral part of the insoluble matter consists almost entirely of the debris of the streets, and the detritus of wheels and horse-shoes. These amount to about 15 grains per gallon; which, in the aggregate, is as nearly as possible 81 tons per day for the whole of the metropolis, or 19 for the city. I am not able to apportion the several constituents of the 116·61 tons of solid matters daily discharged from the city sewers; for there is a difficulty in ascertaining the precise extent of the area drained, and the number of the population; but by taking the whole of the metropolis, the difficulty disappears. Then, of the total amount of 488·5 tons of solid matter contained in the sewage of one day, about 152·60 tons are the ejecta of the inhabitants; 81·08 tons, the pulverized granite and iron from traffic on the roads; 102·04, the saline matter contained in the water supply; and the residue, 152·78 tons, is from trade and manufactures. The total amount of organic matter in all this is about 215·14 tons; of which half is in a state of solution, and the rest is suspended.

"The physical properties of sewage are peculiar, for when it is examined under the microscope, it is found that the clear supernatant part contains a large quantity of amorphous organic matter, with the filaments of various fungi. It swarms with animal life, as beaded *Spirulina*, *Vibriones*, and *Monads;* and soon after exposure to air the higher forms of infusoria appear, as, *Paramecium*, *Vorticella*, *Rotifera*, etc. Besides which, it contains small particles of animal and vegetable tissues, as the fibres of cotton, wool, etc.

"The sediment, which is black and glutinous, consists of the remains of undigested food, as muscular fibre, husk and hair of wheat, the cells and starch of potatoe, and the tissues of cabbage, and other vegetables. It also contains the products of some of the secretions, as yellow biliary matter, intestinal mucus, and the crystals of uric acid and triple phosphate from urine. As in the last case, the living animal forms are numerous; and the vegetable growths are *Oscillatoria*, *Conferva*, *Vegetable spores*, and numerous *Fungi.* The mineral part is composed of the *débris* of the streets, as particles of granite, flint, and carbonate of lime, with a large quantity of black sulphuret of iron. When the sewage has a very unpleasant odour, and is charged with sulphuretted hydrogen, it never exhibits much sign of animal or vegetable life, notwithstanding that it contans an abundance of decaying organic matter. This is the case with the foul contents of the nearly stagnant sewers. But when it is diluted with water, and exposed freely to the air, the bad odour soon disappears, and the higher forms of infusoria are rapidly developed. This is proof of the salutary influence of air and water in promoting the less hurtful kinds of decay. I have noticed the same fact on many occasions, when too large a quantity of sewage has been discharged into a running stream. The insoluble matters settle, and do not obtain a sufficient supply of air or fresh water, to check the putrefactive decomposition, which goes on with great activity: an abundance of foul gas is thus generated, and little or no organic life appears. This happens to the black muddy water on the banks of the Thames, where the only living things are *Monads*, *Vibriones* and *Fungi*. But in the middle of the stream the air and water have so completely destroyed the foul gases, that the highest species of infusoria abound; and the same thing may be observed in every river that receives the sullage of a large town. At first, when the sewage enters the stream, it contains nothing of vegetable life but the simplest fungi, then come higher forms of vegetation, as *Conferva*, *Calothrix nivea*, *Vaucheria*, etc., the last being known by its dirty brown colour and short hair-like structure. Soon after this, as the process of oxydation goes on, and the excess of organic matter is destroyed, higher and higher forms of vegetation appear, and at last the *Anacharis*, *Nasturtium*, *Veronica*, and other aquatic plants abound; and when these are clean and healthy, the sewage is no longer injurious to animal life, for fish will thrive in it.

"The Putrefactive Decomposition of Sewage.—Looking at the enormous quantity of organic matter contained in sewage, it is not surprising that its decomposition is attended with the evolution of a large amount of noxious gas; and when we consider that it is composed of organic matters which have just lost their vitality, and are exceedingly prone to decay; that the solid constituents of it are in a finely divided state, and are therefore in the best form for chemical change; that the proportion of water is just sufficient to hold them in a state of suspension; that the temperature of the sewers is exactly suited for putrefaction; and above all, that the complex mixture is brought into contact with matter already in a state of active decomposition, it is not remarkable that it should at once take on the putrefactive change, and begin to evolve foul gases directly it enters the sewers. Under ordinary circumstances the solid excrements do not ferment in less than three or four days, but here the catalytic influences are so strong that putrefaction begins at once, and it is always of the same kind as that already in progress in the old sewer matter. This tendency to accelerate and direct the decomposition is very remarkable. Its power lasts for weeks after the sewage has ceased to ferment, or it will operate immediately on all kinds of organic substances. Blood, sugar, fæces, urine, and other fermentable bodies, are rapidly changed by it; and they evolve compounds of a most offensive character. Common sugar, instead of being fermented into alcohol, is converted into lactic acid, which smells like putrid pigs'-wash: then it passes into butyric acid, and gives off hydrogen and carbonic acid, with the odour of rancid butter and human perspiration. The same thing occurs to other vegetable matters that are susceptible of change into sugar; and although they do not contain nitrogen, or sulphur, or phosphorus, which are the usual stink-producing elements, yet they evolve compounds that are in the highest degree offensive. Albuminous matters produce ammonia, carbonic acid, and sulphuretted hydrogen, all of which are found in the atmosphere of sewers. But besides these there are many intermediate products of decay which have not been fully investigated; and little is known of the part played by atmospheric air in the sewers beyond this, that when oxygen intervenes in the molecular movements, it gives birth to mineral products, as water, carbonic acid, the sulphates, phosphates, and nitrates, which are the final products of decay. Its influence, therefore, is most salutary, and ought not to be disregarded. Experiment has also shown that the oxydising power of the air is promoted by water, by porous substances, and by the fixed alkalies.

"In seeking to know what part of the sewage it is which undergoes decomposition, I have ascertained that it is not the liquid part which continues to ferment, but the solid, and keeps up the putrefactive action for months, evolving large quantities of ammonia, sulphuretted hydrogen, marsh gas, and carbonic acid. It is the sedimentary matter, therefore, which is the chief cause of the offensive effluvium, and to this I shall direct attention hereafter.

"The Nature of the Sewer Gases.—This part of the inquiry is of great importance; for very little can be really done in the way of providing a remedy for the sewer miasms, until something is definitely known of their nature and composition. Hitherto this subject has been treated quite empirically; and the suggestions which have been made from time to time for ventilating the sewers, and destroying the foul gases, have had no foundation in a right knowledge of the things to be dealt with. The history, therefore, of these schemes is a history of unprofitable failures, and is of no practical value, beyond that of informing us what cannot be accomplished.

"I have endeavoured to procure the knowledge which is wanted from three sets of investigations, viz.:—1st, from inquiries into the composition of the gases dissolved in sewage; 2nd, from an analysis of the gases evolved during its putrefaction; and 3rd, from an examination of the sewer air itself. Each of these modes of investigation has furnished valuable results.

"1st. If the clear liquor of sewage is heated to the boiling point, it evolves all the gases which were in solution. They consist of carbonic acid, sulphuretted hydrogen, ammonia, marsh gas, and nitrogen. The quantity of them varies from about 32 to

76 cubic inches per gallon; and the proportion of carbonic acid varies from 36 to 72 per cent.; the sulphuretted hydrogen from 0·9 to 3·1. Thames water, near to the shore at low tide, contains the same gases in nearly the same proportions.

"2nd. While the insoluble part of sewage is fermenting, it gives off a large quantity of gas, the amount of which is influenced by the proportion of organic matter in the deposit. In the case of one of the worst of the city sewers, where the sewage contained 128·8 grains of organic matter per gallon, the quantity of gas evolved during the first week of putrefactive decomposition amounted to 3 cubic inches per hour. From that time to the end of the third week, it amounted to 1·56 cubic inch per hour; and so it gradually fell until the end of the ninth week, when the quantity was only 0·17 per hour. The average of the whole time was about 1·2 cubic inches per hour; and, as in the last case, the gases were composed of carbonic acid, carburetted hydrogen, ammonia, sulphuretted hydrogen, and azote—the proportions being about 72 per cent. of light carburetted hydrogen, 17·7 of carbonic acid, 10·2 of nitrogen, and 0·08 of sulphuretted hydrogen, with a notable proportion of ammonia.

"In my visits to the city sewers, I have remarked that the same gases are abundantly evolved whereever the sewage becomes stagnant or nearly so. The thick slushy matter in the sewer of Catherine-wheel Alley, for example, furnished me with a large supply of gas. It was bubbling up from the deposit; and was so highly charged with light carburetted hydrogen that it could be ignited with ease; and in the act of burning, it would frequently set fire to the neighbouring bubbles, and produce a sheet of flame that would extend for some distance along the surface of the sewage. The gas contained 68 per cent. of inflammable air, 17·6 of carbonic acid, 14·1 of nitrogen, and 0·2 of sulphuretted hydrogen.

"Large quantities of gas are also generated in the sewage deposit which has been deodorized with lime. This is noticed in the subsiding tanks of the works at Tottenham and Leicester; but, in this case, the gas is composed almost entirely of light carburetted hydrogen, there being but little carbonic acid, and not a trace of sulphuretted hydrogen.

"The amount of ammonia contained in the liquid is always considerable. It ranges from 3 to 15 grains in the gallon of ordinary sewage, and from 15 to 41 in that of the nearly stagnant sewers.

"In addition to all these there are other volatile compounds which have not yet been isolated—compounds which give to the sewage its peculiar odour, and perhaps also its poisonous action. One of them is evidently of an alkaline nature, and is nearly allied to ammonia. It is easily procured by distilling the liquid; and its properties are highly offensive. Dr. Odling has examined it, and has noticed that it contains a large amount of carbon. My own analyses, like his, have given with the impure muriatic salt about 6·61 per cent. of carbon; and the platinum compound about

41·73 per cent. of metal. Dr. Odling thinks it may be the conjugate body *ethylamine;* but it is rather premature to speculate on its nature, before there is some certain knowledge of its composition.

"3rd. The analyses which I have made of the atmosphere of the city sewers, show that the air is deficient in oxygen; and that it contains an excess of nitrogen, carbonic acid, and ammonia. The proportion of sulphuretted hydrogen is rarely so large as to be discoverable in definite quantity; for although I have never failed to recognise its presence, yet the amount of it could not generally have been greater than one part in 60,000 of the air. Marsh gas is also present in minute quantity; and there are also organic compounds, which, as I have said, have not yet been isolated. Their existence is known by the formation of water and carbonic acid, when the air, completely deprived of these matters, is passed through an ignited tube filled with metallic copper. The quantities of the products vary considerably, but they are never such as to indicate the same proportions of carbon and hydrogen as are found in marsh gas.

"By another mode of proceeding, I have been able to condense the organic vapour from the sewer atmosphere, and obtain it in a liquid form. A glass globe filled with ice, was suspended from the crown of a sewer, and the cold thus produced has, in the course of a few hours, caused the condensation of aqueous vapour, and with it the organic matter contained in the atmosphere. In this way, on three separate occasions, I have obtained from 1½ ounces to 4 ounces of liquid, the properties of which were as follows. It had a turbid appearance, and contained minute flocculi of organic matter. Its odour was very disagreeable, like that of bad sewage. Its re-action was alkaline; and it contained free ammonia, with a large quantity of sulphate of ammonia,—a compound derived from the oxydation of sulphuretted hydrogen. Nitrate of silver, and chloride of gold, were quickly reduced by it; and a drop of sulphuric acid added to the liquid and evaporated, gave a black organic residue. All these are proofs of its containing organic matter.

"When the flocculi were examined under the microscope, they were found to be composed of amorphous organic debris, with myriads of *Vibriones* and *Monads.* There were also present the higher forms of infusoria, as *Spirulina, Vorticella, Paramecium,* etc., with the ovules of vegetables, and the filaments of *Fungi* and *Conferva.*

"Parent-Duchâtelet collected the condensed water from the sewers of Paris, and he also found that the liquid had a peculiarly faint odour, and an alkaline re-action; and that on allowing it to stand, it quickly began to putrify.

"These results prove that the atmosphere of the sewers is charged with decomposing organic matter, and with the germs of living organisms. It is also deficient of oxygen, and contains an undue proportion of carbonic acid, nitrogen, and ammonia, with a notable quantity of sulphuretted hydrogen. In the case of the city sewers, these gases are not present in very large quantity; but they may accumulate to a dangerous extent, as sometimes happens in the air of stagnant sewers. When, for example, the sewers of Amelot, De la Roquette, Saint Martin, and others of Paris, were cleansed in 1826, they were found to be highly charged with these foul gases, as with from 0·2 to 1·2 per cent. of sulphuretted hydrogen, and from 0·5 to 3·4 per cent. of carbonic acid; and the fatal accidents which have occurred in the sewers of this metropolis are a sufficient proof of it.

"PROPERTIES OF THE SEWER GASES, AND THEIR EFFECTS ON THE ANIMAL SYSTEM.—It is hardly possible to form a correct opinion of the injurious qualities of the sewer miasms without a previous acquaintance with the properties of the individual constituents; and foremost in the rank of these is *sulphuretted hydrogen.* This gas is constantly produced during the putrefactive decomposition of sewage. It is known by its peculiar odour, which is sufficient to discover it, when it is diluted with 10,000 times its bulk of air. The gas is a little heavier than atmospheric air, in the proportion of 1,179 to 1,000; but its diffusive power is so great that the increase of weight has little or no influence in making it occupy a low level. In a pure state it is highly combustible, and burns with a pale blue flame,—the products of combustion having the odour of burning sulphur. When it is diffused through atmospheric air, it may form an explosive mixture: and if the proportion of air is less than 7½ times the bulk of the gas, the hydrogen only is burnt, and the sulphur is set free; but if there is a larger supply of air, as from 8 to 9 times its bulk, then it is completely consumed, and it forms aqueous vapour and sulphurous acid.

"Water and the fixed alkalies absorb it freely; in fact the latter take it up in large quantity, and render it comparatively innocuous, Chlorine and chloride of lime destroy it by combining with the hydrogen and setting free the sulphur. Almost all the common metals are discoloured by it; indeed, the salts of lead and silver are so quickly blackened, and are so sensitive of its action, that they indicate the presence of the gas when the atmosphere does not contain more than one part in about 100,000: these, therefore, are the tests for it.

"Sulphuretted hydrogen is exceedingly poisonous; it will destroy life under all circumstances, whether the gas is inhaled, or absorbed through the skin, or injected into the cavities and tissues. Dupuytren and Thenard have found that one part of the gas in 1500 of air will kill small birds immediately, and that one in 290 is fatal to rabbits and dogs in a few minutes. Horses are killed by an atmosphere containing one part of it in 250 of air; but much less is hurtful if it is breathed for any length of time.

"Dr. Barker in his recent inquiries into the action of this gas on the animal system, has ascertained that one part of it in 1800 of of air, is immediately fatal to birds, and that so small a quantity as one in 2112 of air will sometimes kill them in about a minute and a half. Larger animals are not so easily affected, although dogs are quickly killed by a mixture containing one part of the gas in 210 of air, and more slowly by one in 500. My own experiments are to the same effect, for I have found that one part of gas in 500 of air is soon fatal to rabbits; and I have no doubt from the energy of its action that such a proportion would be highly dangerous to all classes of animals. These facts make it difficult to understand the accounts given by Parent-Duchâtelet and others, of the thriving of rats and mice in sewers, which have an atmosphere composed of from one to two per cent. of the gas; and I am inclined to think that the means which were at the disposal of his colleague, Gaultier de Claubry who examined the air, were not sufficient to determine the actual proportion of the mephitic gas.

"Observation has likewise shown that when the gas is diluted with very large quantities of atmospheric air, its action is slowly but surely deleterious. Of this, there are many examples. When the men were engaged in cutting through the bed of the river, for the construction of the Thames Tunnel, they suffered severely from the effects of the gas, although the proportion of it in the air was hardly to be discovered by lead-paper, and could not, therefore, have exceeded one part in 100,000. It is true that it sometimes came in gushes from the fissures of the mud, but the quantity was rarely sufficient to be recognisable by its odour. Strong and robust men were, however, reduced to a state of extreme exhaustion, by breathing it for a few months; and several of them died from it. The symptoms with which they were affected were giddiness, nausea, or actual sickness, and great debility. The men became emaciated, lost their appetites, and fell into a state of low fever, from which, in several instances, they did not recover. Chloride of lime, and other prophylactics, were used; but the evil did not entirely cease until the tunnel was opened from end to end, and free ventilation established.

"According to Dr. Taylor, another remarkable instance of the same kind of poisoning occurred last summer at Clayton Moor, near Whitehaven, where there is a row of small cottages built on the refuse slag of some neighbouring iron-furnaces. The houses are occupied by the workmen and their families, who, for some time, had been annoyed by an offensive smell, which pervaded the lower rooms. Suddenly, however, in the month of June of last year, the smell became unusually offensive; and, in the course of two days, thirty of the inhabitants were made seriously ill by it. The attack was remarkably sudden, as if a poison had been at work. In one of the houses there was a family of seven persons, consisting of the husband and wife, and five children. They retired to rest in their usual health, but in the morning two of them were dead, and the others were in a state of profound insensibility. Before the day was over, another of them died, and in the course of the week a fourth. In a second case, a strong, healthy man came home from his night-work, and went to bed; but an hour had hardly elapsed, when he also was found dead. And in a sixth instance, a child was taken ill in the morning, aud was a corpse at night. An inquiry was instituted for the purpose of discovering the cause of the mischief, and Dr. Taylor came to the conclusion that it was sulphuretted hydrogen, generated by the action of water on the refuse slag, upon which the houses were built. If so, it is a remarkable instance of the poisonous action of this gas; for those who examined the air of the rooms declare that the test of lead-paper failed to show the presence of the poison, except in mere traces—that is, in quantities which could not have been greater than 1 part in 100,000 of air; for such a proportion would have been easily recognised if it had been present. It is unfortunate that an ultimate analysis of the air was not made, for that might have proved the existence of some other gas, perhaps carbonic oxide, which is largely produced in the furnaces, and which might have been wafted from them to the bed-rooms. Those who survived the action of the poison, declared that they were suddenly attacked with giddiness and insensibility; and this was followed by a death-like coma. In some cases there were nausea and vomiting, and in others there was a feeling of oppression and closeness in the air.

"These experiments and observations show that sulphuretted hydrogen gas is a powerful narcotic poison; that in a concentrated state it kills immediately, as with the energy of prussic acid. In a more dilute form it causes death by lingering stupor; and when more diluted still, so as hardly to be discovered by its odour, it prostrates the vital powers, and produces a low fever which may end fatally. Dr. Barker, in his prize essay, has summed up his observations of the effects of the gas in these words:—"The symptoms arising from sulphuretted hydrogen are well marked, and may be considered specific. Vomiting and diarrhœa are the first and most prominent symptoms. The latter is painful; the vomiting is difficult and exhausting; and eventually there is insensibility and entire prostration. When the dose of the poison is at first very large, the prostration and insensibility are immediate. The pathology following such poisoning is also definite. If the death take place quickly, the pathological evidence is that of asphyxia. If the poison is long breathed in a diluted dose, the pathology is modified; the fibrine of the blood is separated, and the heart is slowly clogged up with fibrinous depositions."

"*Carbonic acid* is also produced by the decay of organic matters. It is found in the air of sewers to the extent of 0·5 to 2·3 per cent.; and the gases evolved from the sewage itself contain about 19 per cent. of it.

"This gas is not easily recognised by its sensible properties, for it has no distinct odour, unless it is breathed in a very concentrated state, and then it irritates the nose and throat in much the same way as the fumes of burning sulphur. It is heavier than air in the proportion of 1,525 to 1,000, and its diffusive power is very great. A taper will not burn in it even when the gas is diluted with eight times its bulk of air. This, therefore, is generally the test of its presence; but it is not a good test, for a taper will burn in an atmosphere in which an animal cannot exist,—as, for example, in air containing about 10 per cent. of the gas.

"As in the last case, the gas is a powerful narcotic, and will cause immediate asphyxia, if it be breathed in a somewhat pure condition. Even when it is diluted with four times its bulk of air, it destroys life in a few minutes; and with a much larger dilution it produces giddiness and headache, with a sense of weight and throbbing at the temples. This is sometimes followed by slight delirium, and then by an irresistible desire to sleep, the stupor of which passes slowly into coma. According to M. Varin, these effects are produced by the continued inhalation of air charged with about 2 per cent. of the gas; and MM. Peclet and Leblanc assert that animals are affected by an atmosphere containing only 1 per cent. of it. Even so little as 0·5 per cent. is said to be injurious, if it is breathed for a long time.

"If the gas has been produced at the expense of the oxygen of the air, as happens in sewers and crowded rooms, then the effects are more strongly marked; for under these circumstances as little as 3 per cent. will quickly destroy life. Expired air contains from 3 to 5 per cent. of the gas; and the tragedies of the black-hole at Calcutta, and the round-house of St. Martin's, are examples of the terribly fatal power of such an atmosphere: even the proportion of from 1·5 to 2 per cent., will cause almost immediate distress, and a feeling of suffocation. These were the proportions found by Dr. Bence Jones in the dormitories of a metropolitan workhouse, where the effects of the vitiated air were remarkably unwholesome; and, to quote another familiar case, in the atmosphere of the Wellington barracks, where the sickly household troops are lodged, Dr. Roscoe ascertained that the quantity of carbonic acid at night ranged from 0·12 to 0·14 per cent. In a crowded theatre it does not exceed 0·32 per cent.; and yet there are few persons who have not experienced the depressing influence of such an atmosphere. What shall we say, therefore, of the sewer gases, which contain from 0·5 to 2·3 per cent. of it?

"*Ammonia* is another constituent of the sewer air; and is a product of putrefaction. It is known by its peculiar odour, and alkaline reaction. It is lighter than the air in the proportion of 590 to 1,000. The gas irritates the mucous membrane of the nose and eyes; and in some cases it is found in sufficient quantities to produce ophthalmia, and inflammation of the lungs. Parent-Duchâtelet speaks of the first of these as a common effect on the nightmen of Paris, but we do not observe it in this country. When ammonia is inhaled in a concentrated state, it produces immediate asphyxia; when it is somewhat diluted with air, its action is chiefly on the lungs; and when it is more diluted still, and is breathed for a considerable time, it liquifies the corpuscles of the blood, and produces the symptoms of typhoid fever. Dr. Richardson has lately inquired into this matter, and he finds, from experiment, that the continued action of the gas, even when it is largely diluted with air, is peculiarly injurious to the animal economy, 'the tongue becomes dry and dark; there is an involuntary action of the muscles, varying from a mere twitching to violent convulsions; there are insensibility, extreme sensitiveness to sound, obscurity of sight, and ultimately, if matters are pushed far enough, death by coma. The morbid anatomy is equally demonstrative. The blood is dark and fluid; the serous membranes show petechial spots, and the tissues are softened.'

"Dr. Barker's experiments have confirmed these observations; and they prove that the continued inhalation of atmospheric air, charged with only a small quantity of ammonia, is dangerous to health.

"But there is another property of ammonia which is more dangerous still: it is that of conveying the less volatile products of putrefaction into the air. In all probability, it is the purveyor of the miasms of infected districts, as it is known to be of the fœtid compounds of animal and vegetable decomposition. It was the agent which gave volatility to the putridities of the Thames during the hot weather, and it is the medium by which the more offensive matters of coal gas are held in suspension. Nor is it less powerful in diffusing the sweet odours of plants, and the subtle constituents of many perfumes. It may therefore act for good as well as for evil.

"*The volatile compounds of ammonia, with carbonic acid, and sulphurated hydrogen*, are also injurious. The first act like the alkali itself, and the second like sulphuretted hydrogen. I have found that one part of hydrosulphate of ammonia in 1,000 of air will kill birds almost instantly; and one in 500 will kill rabbits. Dr. Barker has noticed that the symptoms produced by it, even in small quantity, are vomiting, diarrhœa, muscular debility, and quick respiration. The surface of the body becomes hot, and then cold; the tongue protrudes, and looks livid and dry; there is constant twitching of the muscles, and jerking of the limbs; the pulse is quick and feeble; and death follows within a short time, even if the animals are removed from the poisoned atmosphere, and placed in the open air. In an experiment by Dr. Richardson on a dog, the symptoms were those of low fever; and after death, he found the same kind of ulceration in the intestines as is noticed in the typhoid form of the malady.

"*Light carburetted hydrogen, or marsh gas*, is also found in the atmosphere of sewers, but it rarely occurs in such proportion as to be dangerous; now and then, however, it accumulates, as it does in coal mines, and becomes explosive.

"The gas is a product of putrefaction, and it is likewise formed by the action of organic carbon on the elements of water. It escapes freely from the mud of stagnant ditches, and, as I have already said, from the solid deposit of the sewers. The gas has no odour, and therefore cannot be recognised by its sensible properties. It is about half as heavy as air, the specific gravity of it being 557·6; and it is very combustible, burning with a yellow flame. When it is largely diffused through the atmosphere it forms an explosive mixture, the violence of which is dependant on the proportions of the air and gas. If there is one volume of gas to about 9·5 of air, the explosion is most powerful; but the proportions on either side of this are not so violent; for the shock becomes gradually weaker and weaker until, with one of gas to 14 of air, or 4 of gas to one of air, the mixture does not explode at all. When the combustion is perfect, the products are carbonic acid and aqueous vapour, with the residual nitrogen of the air—the volume of carbonic acid being the same as that of the gas consumed. Such an atmosphere is therefore dangerous to life, not only because of its deficiency of oxygen, but also because of its containing so large a quantity of carbonic acid; and this is the thing to be feared after an explosion in a sewer or coal mine.

"Marsh gas has but little action on the animal body, unless it is breathed in a pure form, and then it causes the general effects of anæsthesia. Miners are often obliged to work in an atmosphere containing from 8 to 10 per cent. of the gas, but they experience no ill effects from it, until the proportion rises to about 20 per cent., and then they feel giddy, with a sense of weight upon the forehead.

"*Coal gas* is likewise present in the sewers: it is not formed there, but escapes from the street mains. The quantity which is let loose in this manner is enormous. Gas engineers say that from 12 to 35 per cent. of all the gas manufactured in London is lost. Now supposing that of this, the leakage amounts to 5 per cent., which my friend Mr. Wright informs me is about the quantity; then as much as 386,400,000 cubic feet of gas escape into the public ways of this metropolis every year, or rather more than a million cubic feet every day; and in the city it amounts to about 25,000,000 cubic feet per annum, or nearly 70,000 cubic feet per day. Most of this must find its way into the sewers, and therefore is a matter of some importance.

"The chief constituents of coal gas are hydrogen and light carburetted hydrogen. The former amounts to about 40 per cent. of the gas, and the latter to 45. The other constituents are about 7 per cent. of carbonic oxide, 2 of nitrogen, 1 of carbonic acid, and 5 of the condensable hydrocarbons. Besides which there are always traces of ammonia, bisulphuret of carbon, and coal tar.

"The principal danger from this gas is, its inflammability, and its property of forming an explosive mixture with atmospheric air.

"The force of the explosion varies with the mixture, and with the volume that is fired. Theoretically, it is known that marsh gas in exploding, exerts a pressure of 38 atmospheres, hydrogen 26·5, and carbonic oxide 22·6. The force of the other hydro-carbons varies from 44 to 88 atmospheres. My experiments on this point have not been numerous, but they indicate an explosive power of about 30 atmospheres, for ordinary coal gas.

"The physiological properties of coal gas are more marked than those of light carburetted hydrogen; for the condensable hydro-carbons are endowed with great activity as poisons. When, therefore, the gas is inhaled in a concentrated state, it quickly produces insensibility, which is followed by coma and death. An atmosphere containing from 7 to 12 per cent. of gas will kill rabbits and dogs in a very short time; and M. Tourdes found that even a thirtieth or fiftieth part of it in the air, would act injuriously on animals. This proportion causes in the human subject, nausea and headache, with great depression of the vital powers; and if the inhalation is continued for a long time, it produces giddiness and insensibility. Dr. Taylor has collected the records of seven cases of death from the action of coal gas, and it is probable that the air was not charged with more than 8 or 9 per cent. of it in any of them.

"As to the properties and effects of the organic vapour which is contained in sewer gases, I cannot speak with certainty, except that it is matter in a state of active decomposition; and experience has long since decided that matter in this condition has power to disturb the equilibrium of other organic molecules, and to propagate to them its own state of decay. When this occurs in the living animal body, it is productive of the most terrible consequences. Our ignorance of the nature of this organic vapour is not surprising, when we consider that we are equally uninformed of the composition of the subtle miasms and putridities which abound in the air of infected districts, and in the vapours of organic decomposition. Guntz tried to catch the volatile matters of putrid flesh, but he only obtained a stinking liquor that could not be analysed. Moscati condensed the miasm of the pestilential rice fields of Tuscany, but the liquid which he procured defied investigation, notwithstanding that it was so charged with organic matter, that it quickly putrefied. Rigaud did the same with the atmosphere of the marshes of Languedoc, and Boussingault with the air of some of the worst districts of Paris; but their labours were fruitless. Even the wards of a hospital crowded with cholera patients, and the fœtid dormitories of our household troops, and the no less unwholesome dens of the city poor, have been vainly searched for the occult molecules, which are endowed with so much virulence. All that we know of them is that they will reduce the salts of silver and gold, and blacken sulphuric acid; that they contain the elements of organic matter in a state of active change; that they quickly putrefy; and that they form a nidus for the growth of the lowest forms of aimated beings.

"Sometimes these miasms are odourless, but in the case of the sewer gases, it is the organic vapour which gives them their peculiar smell; for when the sulphuretted hydrogen is entirely renewed, there are still the characteristic stinks, which have been so accurately described by Parent-Duchâtelet.

"What are the Dangers of the Complex Sewer Gases themselves? Experience has shown that they are of two kinds; namely, the dangers which are incidental to the poisonous action of the gases; and those which arise from their explosive property.

"As to the first of these, it must be evident, from what we know of the composition of the gases, and the properties of the individual constituents, that the complex miasms must be exceedingly hurtful to the animal economy. Observation has confirmed this, by proving that they are among the most active poisons. One breath of the undiluted gases will destroy life immediately; and even when they are mixed with a rather large quantity of atmospheric air, they quickly cause asphyxia, or narcotism and death. Those who are exposed to their influence are suddenly deprived of consciousness: they fall powerless, as if they were shot; the lips become livid; the face turgid; bloody froth oozes from the mouth; the surface of the body gets cold; the action of the heart tumultuous and irregular; the breathing difficult and spasmodic; and the sufferer dies in convulsions, or lingers for a short time in deep and irrecoverable coma. In smaller quantity the miasms are less active, but not less certainly injurious; for they produce nausea or actual sickness, vertigo, delirium, and gradual insensibility. The muscles of the face and chest are convulsed; and the action of the heart is feeble and fluttering; the skin acquires a death-like coldness; and the respiration is irregular and catching. In this state the danger is imminent, and if means are not promptly taken for recovery, the patient dies in a state of coma. If the gases are still further diluted with atmospheric air, they produce a general prostration of the vital powers. The appetite fails; the bowels become disturbed; diarrhœa of a chronic character sets in; and the sufferer is either worn out by exhaustion, or he falls into a state of low fever, from which it is difficult to raise him.

"I need not weary you with examples of the acute forms of poisoning by these gases, for they are but too well known. It is, however, necessary for my purpose that I should mention a few cases of chronic poisoning, in which the effects were produced by the inhalation of very small quantities of sewer miasms. One of the most remarkable instances of this is recorded by M. D'Arcet. He states that there was a small lodging in Paris, consisting of a bed-room and ante-room, which had been successively tenanted by three vigorous young men, each of whom died within a few months of his occupying the place. D'Arcet was requested to examine the rooms, and ascertain the cause of the evil, He found that a pipe from a privy in the upper floor ran down by the side of the wall near to the head of the bed where the inmates slept. The pipe was unsound, and the wall was damp from leakage of the soil into it; but there was no perceptible smell in the room when

D'Arcet examined it: nevertheless he had no doubt that the deaths of the former occupants were referable to the emanations from the wall. The pipe was, therefore, repaired, and from that time the unwholesomeness of the place was cured.

"In August 1831, twenty-two boys living at a boarding school at Clapham, were suddenly seized with alarming symptoms of irritation in the stomach and bowels, with twitchings of the muscles of the arms, and excessive prostration of strength. Another boy had been similarly attacked three days before, and he died in twenty-five hours: one of the others died in twenty-three hours. A suspicion of accidental poisoning naturally arose, and the various utensils, and articles of food used by the family were examined, but nothing of a deleterious nature was found. The only circumstance which appeared to explain the accident was, that two days before the first child was taken ill, a foul cesspool had been opened, and the matter of it diffused over a garden adjoining to the children's play-ground. This was considered to be the cause of the disease; and the opinion was formed not only by the medical attendants, but also by Drs. Latham, Chambers, and Pearson, who personally examined the whole of the particulars.

"The third instance to which I shall refer is of more recent date, and has been a subject of considerable discussion. In the month of August 1852, an attempt was made to drain the town of Croydon by means of small stoneware pipes, which were not only of insufficient size but were imperfectly cemented together. The consequence was that a large quantity of the sewage escaped into the earth, and drained away to the neighbouring ditches. This became a subject of great annoyance; and in a short time it produced an alarming outbreak of fever, diarrhœa, and dysentery.

"In the report from the Poor Law Commissioners on the sanitary condition of the labouring population of Great Britain there are many examples of the morbific action of sewer and cesspool gases. There is one case which is remarkable for its significance. On the north side of a street in Derby there are fifty-four houses, all of the same description, and inhabited by the same class of persons. Six of these houses, in the very centre of the row, became the abodes of fever; and of sixteen persons attacked with the disease, five died. The fever was nowhere else in the row; and on inquiry it was found that these, and these only, were exposed to the action of sewer gases, and to the miasms from cesspool matter which had soaked into the soil.

"The Remedy for the Sewer Miasms. This is the great question which you have submitted to me for consideration, and the preceding facts show clearly enough that it is an important question. I am far from thinking with your engineer that the mischief occasioned by sewer gases is not of such magnitude as to be worth a remedy that may cost sixpence a head to the population; nor do I believe that if you had temporised with the matter, and had yielded to the demand on the part of the public, to close up the ventilating gratings, which are now so offensive, and had thus turned the foul gases into the house drains, the nuisance would have been regarded simply as a domestic evil, for which the cure was to be sought privately and individually by those who felt the annoyance: in fact, such a proceeding would have been unworthy of the trust which is reposed in you as the custodians of the public health, for it would have been a matter of life or death to the great bulk of the community.

"At all times attempts have been made to destroy or neutralise the offensive products of decomposition; and the simplest way of doing it has been by the use of another scent—a perfume or volatile oil, which would cover or mask the offensive body. These were the correctives employed in religious worship. They entered into the composition of the ointments of the high-priest, and the incense of the altar; and to this day they have enjoyed a reputation and general popularity which they have not deserved; for their action is not on the putrid product, but merely on the sense of smell, which they blunt to the action of the offensive vapour. In the middle ages, when the plague, the black-death, and sweating sickness, and pestilential fever, desolated the cities of Europe, immense importance was attached to the use of perfumes: fumigations with costly spice and rich smelling oriental drugs were largely used in the houses of the rich, but with no good effect. The ancients also knew the value of fire as a disinfectant; and they also made use of the fumes of burning sulphur.

"But the right knowledge of the action of disinfectants and deodorisers dates from a very recent period; for so late as the year 1773, Guyton Morveau, one of the best chemists of France, thought that the vapours of muriatic acid were the most powerful of disinfectants; and later still, in the year 1803, Dr. Carmichael Smith obtained a grant of £5,000 from parliament for a suggestion which is nearly valueless, namely, the employment of the fumes of nitrous acid. Chlorine gas was discovered by Scheele in 1774, and soon afterwards its disinfecting properties were noticed by Berthollet; but its use cannot be dated farther back than the beginning of the present century, when Guyton Morveau and Dupuytren first pointed out the great value of it as a disinfectant; and even then it was not generally employed: in fact, its present popularity dates only from the time that chloride of lime has been largely manufactured for bleaching purposes. Within the last twenty years, almost all the refuse products of the arts, and a great number of special compounds, have been recommended for the deodorisation of sewage, etc. They all act in one of two ways: they either give stability to the organic matter, and so check its tendency to decay, or they operate on the putrid vapours, and destroy their offensive properties. This they do either by fixing the effluvium, and forming compounds which are inert, or by breaking up the putrid molecule, and changing its nature, or by expediting the process of decay, and hurrying it on to the last stage of oxydation.

"Those substances which give stability to organic matter are properly called *anti-septics* or *anti-putrescents*. They have always been more popular than any of the second class of deodorisers, because of their importance in the arts. Salt, sugar, vinegar, creasote, and the empyreumatic oils of wood, peat, coal, etc., are examples of this class; so also are chloride of zinc, sulphate of copper, and corrosive sublimate—substances which have been respectively patented by Sir William Burnett, Mr. Margary, and Mr. Kyan. Alum, and the astringent matter of many vegetables, have likewise been used for ages as the means of preserving gelatinous tissues, in the form of leather. None of these, however, except the chloride of zinc, is applicable to the case before us; and that operates more as a deodoriser than an anti-putrescent. In fact, as I have already stated, the matters of sewage are always in a high state of decomposition, and cannot therefore be treated with much advantage, unless the antiseptics are applied to them, before they enter the sewers: and this, I need not say, is altogether impracticable. Even in Paris, where there are special contrivances for such a purpose, it fails because of its utter impracticability.

"Of the second class of substances, namely, the *deodorisers* and *disinfectants,* there are many which deserve notice.

"Those which combine with the putrid gases, and fix them in an involatile form, are the metallic oxides, and their salts, as chloride and sulphate of zinc; acetate and nitrate of lead; sulphate, muriate and pyrolignate of iron; impure muriate of manganese—the refuse of bleaching works; common alum; the fixed alkalies; and the salts of lime and magnesia. Most of these compounds unite with the sulphuretted hydrogen and ammonia of sewage; and so far, therefore, they remove the unpleasant smell of if, but they do not touch the organic vapours. Besides which, they are difficult to apply, and are very costly. In fact, putting aside all the working expenses that would attend their use, the mere cost of the deodorizers alone would range from about £20,000 to upwards of £1,000,000 per annum for the city sewage; and from £1,000,000 sterling to £48,000,000 for the sewage of the whole of London. This, together with the insufficiency of their power, as deodorisers, and the difficulty of applying them while the sewage is within the sewers, deprives them of all practical utility. That power which they do possess, namely, the power of coagulating a great part of the soluble matter of sewage, and favouring the precipitation of the insoluble, can only be applied, with any chance of success, after the sewage has left the sewers; and even then there are but two of them, namely, lime and the superphosphate of lime with magnesia, that can be used with advantage.

"Of the second class of disinfectants, those which act chemically on the volatile matters and break them up, so as to form new compounds, which are inert, the most important are, chlorine, chloride of lime, hypochlorous acid, sulphurous acid, and nitrous acid. The first three of these, namely, chlorine and its oxy-compounds, operate by abstracting hydrogen from the putrid vapours, and perhaps also by decomposing water, and setting the oxygen free to destroy the miasms. Their power is remarkably great, as may be seen by the action of chlorine or chloride of lime on ordinary sewage; eight grains of chloride of lime, or less than an ounce of a solution of chlorine will completely deodorise a gallon of sewage, and the diffusion of a little chlorine through the worst kind of sewer gases is sufficient instantly to deprive them of their offensive odour. Nevertheless, chloride of lime is a costly agent; for, if it be used in the proportion of only eight grains to the gallon, it will cost nearly £57,000 a year, for the deodorisation of the city sewage, and nearly £237,000 for the sewage of all London. As for the gas itself it is almost impossible so to apportion and manage the diffusion of it in the sewers as not to have either the chlorine or the sewer gases in excess.

"Sulphurous acid and nitrous acid are still less manageable. Besides which they are exceedingly costly, and are not nearly as powerful in their action as chlorine. Like chlorine, however, they disorganise the putrid molecules, and decompose the hydro-sulphur compounds, and fix ammonia; but, like it also, they are powerful irritants, and could scarcely be let loose into the sewers without danger to the workmen.

"The last of the disinfectants are those which expedite the process of decay, and cause the putrid matters to combine with oxygen, and to become inert. Of this class there are two members, namely, those which act chemically and supply oxygen of themselves to the offensive compounds; and those which merely facilitate oxydation by their physical properties. The manganates and per-manganates of potash are the best examples of the first class. They contain a large proportion of oxygen, which they freely give up to putrid organic matter, and so destroy it. These compounds have been patented by Mr. Condy, of Battersea, and he supplies them in a state of solution of various strengths. That which I have used in my experiments contained nearly 6 per cent. of the per-manganate, and could be supplied at a shilling a gallon. One hundred and fifty drops of it were sufficient to deodorize a gallon of ordinary sewage; but the disadvantage of it is, that it has no power to destroy the foul gases which have already escaped into the sewer air. Besides this, the cost of the material, even if it were used in the proportion of 150 grains to the gallon, would be about £753,000 a year for the city sewage, or more than three millions per annum for that of the whole metropolis. Looking, however, merely at the chemistry of the subject, it must be admitted that Mr. Condy's solution is a powerful and valuable disinfectant.

"The second of this class of disinfectants are the agents which promote oxydation by a physical property, that is by bringing the putrid matters into contact with atmospheric oxygen. There are three of them, namely, fire, water, and porous solids. The first effects the change by active combustion, and the others by a slower process of oxydation, which is called *eremacausis,* or slow burning. All of them, however, are complete in their action, and are, under different circumstances, more or less manageable and useful.

"The value of fire as a disinfectant is well known, and has been recognised from the remotest time. The sacrificial altars of early nations were the rude methods by which the agent was employed; and so fully did the ancients believe in its salutary action, that in times of pestilence it was often resorted to as the only effective means of purifying the atmosphere. In the popular mind there has always been a notion, that the Plague of London was exterminated by the great fire. Powerful, however, as the agent is, it does not appear to be applicable to the destruction of the sewer gases, notwithstanding that the use of it for such a purpose has always been a favourite idea with every new commission of sewers, and is the basis of most of the amateur schemes of the present day. Mr. Bazalgette has stated, in his evidence before the House of Commons, that putting aside all the difficulties of controlling the course of the air in the main channels of the sewers, and of stopping the leakage from the thousands of openings in the streets, closets, and drains, the mere cost of the fuel for the furnaces would not be less than £80,000 a year, and perhaps it might reach to upwards of £200,000.

"The destruction of the sewer miasms by the agency of water is not quite so unmanageable, and has therefore received attention from many of the leading engineers of the present day. Mr. Bazalgette says of it, that it is the best and only available means of purifying the sewers. As to its salutary action there can be no doubt, for its power as a disinfectant, in the presence of atmospheric air, is manifested in every river in the kingdom. Wheresoever the putrid refuse of a town mixes with a large volume of fresh water, there the process of oxydation is quickly carried out, and the offensive matters are rendered innocuous. Even the river Thames, except at a peculiarly dry and hot season, finds within itself a means of purification which is quite equal to the contaminating influence of the soluble organic matter that flows into it. This is effected by the physical power which water possesses of transferring oxygen from the atmosphere to the putrid products; and this power is so great that it will even destroy the soluble organic constituents of ordinary sewage without further dilution with water.

"I cannot inform you very accurately what quantity of water is necessary for the purpose of disinfection. Already there is a daily supply of about thirty-one gallons to each of the inhabitants of this metropolis; but this is evidently not sufficient to cleanse the sewers; for independently of the existence of a putrid atmosphere within them, there is the stronger evidence of their foulness in the condition of the sewage which is discharged immediately after a heavy fall of rain. And even if it could be determined precisely what amount of water would effect the purpose, there is still the difficulty of distributing it so as to scour out all the channels, to the very smallest; for this could not be accomplished without special contrivances for delivering the water at the head of every drain. I do not, therefore, see much prospect of success in this mode of dealing with the subject.

"The last means of destroying the offensive matters of sewage is by the agency of a porous solid; and this may be applied either to the sewage itself, or to the gases which are evolved from it. The best examples of such an agent are common clay and charcoal. Both of them operate in the same way,—namely, by condensing the putrid vapours within their pores and upon their surface, so as to cause them to unite with atmospheric oxygen: they produce, in fact, a species of slow combustion, by which the miasms are gradually consumed. To effect this, however, there must be free access of atmospheric air: hence it is these substances have but a limited power of deodorization when they are mixed with the liquid sewage, or are so overcharged with water as to be incapable of absorbing oxygen.

"Every one is familiar with the deodorizing power of common earth: in fact, the graveyards of every city testify of the enormous quantities of organic matter that can be disposed of through its agency; and no one who has witnessed the rapidly deodorizing power of clay, when sewage or night-soil is distributed upon the land, can doubt its efficacy. The Chinese have long taken advantage of this power; for they mix night-soil with about one-third of its weight of fat marl, and knead it into cakes, which are common articles of commerce. In practice, here also it is found that a ton of clay will deodorize about three tons of the solid matter of sewage. But powerful as this action is, it is not applicable to liquid sewage. Even in the case of charcoal, which is a much more energetic deodorizer than common clay, the power is speedily lost when it is mixed with fluid refuse. Dr. Hofman found that four cubic feet of charcoal began to lose their power of deodorization when about seventy-eight gallons, or rather more than three times their bulk, of sewage had passed through them: and Mr. Blyth's experiments are to the same effect. To this I may add that the cost of this mode of deodorization would be upwards of £230,000 a year for the City sewage, or nearly a million for the sewage of all London; and even then the remedy would be but imperfect, to say nothing of the fact that it would contribute largely to the solid matters already in the sewers.

"The most effective way of using charcoal as a deodoriser, is to take advantage of its power of absorbing the putrid miasms when they are in a vaporous condition. This power is remarkably great. It was noticed by Saussure, as far back as 1814, that charcoal took up from 1·75 to 90 times its bulk of various gases. Count Morozzo had also observed the same fact, and had directed attention to it; and later still, Messrs. Allen and Pepys found that different kinds of charcoal had different powers of absorption. But it is only very recently that we have been well informed of this matter; and we owe our knowledge of it to the researches of Dr. Stenhouse, who, in 1853, had his attention directed to the fact that when the dead bodies of large animals are covered with a layer of charcoal, they putrefy without evolving any unpleasant odour, notwithstanding that they are kept for many months.

"These results suggested the use of charcoal as a respirator and an air filter; and soon after Dr. Stenhouse proposed it as a purifier of the foul gases which escape from the street gullies, the sewer ventilators, and the drains of private houses. One of his air filters is, I believe, in action in the justice-room of the Mansion House, and another in the justice-room of Guildhall; and Dr. Stenhouse reports that their operations have been successful and continuous for a long time.

"I have myself repeated some of Dr. Stenhouse's experiments during the last twelve months; and have ascertained that the offensive gases from a close cesspool are completely deodorized by passing them through a small box containing about 36 cubic inches of coarsely powdered peat charcoal. I have had this in continual action for about three months; and although the charcoal has not been renewed, yet it does not show any sign of derangement or loss of power.

"All kinds of charcoal, however, are not equally valuable for this purpose. Dr. Stenhouse found that wood charcoal and peat charcoal are the most effective. Mr. Blyth's experiments at the Board of Health, and the inquiries made in my own laboratory by Mr. Fewtrell, are to the same effect. The cause of this superiority is doubtless due to the great porosity of vegetable charcoal. Liebig states that the pores in a cubic inch of beech-wood charcoal must at the lowest computation be equal to the surface of 100 square feet; and several other chemists have estimated it at more than double this amount. Hence the extraordinary physical power of wood charcoal in absorbing and condensing gases and vapours within its pores; so that when it is exposed to an atmosphere containing the putrid products of decomposition, it absorbs and oxydises them by a species of combustion that is as effectual as if they were passed through the ignited coals of a furnace.

"Now in making a practical application of these facts it is manifest that we have in common wood charcoal a powerful means of destroying the foul gases of sewers. How it is to be applied is fortunately a question of but little embarrassment; for let the sewers be ventilated as they may, either by open gratings in the street, or by the rain-water pipes of the houses, or by the pillars of the gas lamps, or by tubes carried up at the landlord's expense from the drains of every house, or by especial shafts in the public streets; in fact, let the gases go out of the sewers how they will and where they will, you have but to place a small box containing a few pennyworths of charcoal, in the course of the draft, and the purification of the air will be complete."

